# Influenza A and B Viruses but Not MERS-CoV in Hajj Pilgrims, Austria, 2014

**DOI:** 10.3201/eid2104.141745

**Published:** 2015-04

**Authors:** Judith H. Aberle, Theresia Popow-Kraupp, Peter Kreidl, Hermann Laferl, Franz X. Heinz, Stephan W. Aberle

**Affiliations:** Medical University of Vienna, Vienna, Austria (J.H. Aberle, T. Popow-Kraupp, F.X. Heinz, S.W. Aberle);; Federal Ministry of Health, Vienna (P. Kreidl);; Sozialmedizinisches Zentrum Süd-Kaiser-Franz-Josef-Spital, Vienna (H. Laferl)

**Keywords:** influenza virus, influenza, humans, MERS-CoV, Middle East respiratory syndrome coronavirus, coronavirus, viruses, Saudi Arabia, the Hajj, pilgrimage, Hajj pilgrims, Austria

**To the Editor:** The World Health Organization recommends that persons who return from pilgrimages to the Middle East with acute severe respiratory infections be tested to determine the cause of infection; the aim is to identify infections with the Middle East respiratory syndrome coronavirus (MERS-CoV), which have been occurring in Saudi Arabia since 2012. Each year, >2.5 million persons from >180 countries, including 240,000 pilgrims from Europe, participate in Hajj, the Muslim pilgrimage to Mecca, Saudi Arabia. The gathering of mass numbers of persons during the Hajj increases the risk for the spread of respiratory infections among participants, and this risk has raised global concern that travelers returning from this pilgrimage could contribute to the international spread of MERS-CoV. During the 2012 and 2013 Hajj and Umrah (a minor pilgrimage) pilgrimages, no MERS cases in pilgrims were reported (*1*,*2*). However, in 2014, cases of MERS-CoV infection were confirmed in 2 returning pilgrims in the Netherlands (*3*). The International Health Regulations Emergency Committee advised all countries to improve awareness about MERS-CoV among pilgrims and to conduct surveillance for MERS-CoV among pilgrims during and after Hajj (*4*).

According to data from the International Air Transport Association, Austria received an estimated 68,000 air travelers from Saudi Arabia, Jordan, Qatar, and the United Arab Emirates during June–November 2012 (a period encompassing 1 month before Ramadan and 1 month after the Hajj) (*5*); of these travelers, 1,000 were pilgrims performing the Hajj. Relatively constant travel volumes to Austria on commercial flights out of these countries during 2010–July 2014 have been confirmed by an analysis of air traffic statistics for Austria (A. Herndler, M. Rudolf, pers. comm.). We report on the investigation of illness among Austrian residents just after their return home from the 2014 Hajj pilgrimage, which ended in early October.

As of October 27, 2014, a total of 7 Hajj pilgrims from Austria had sought medical care in different Austrian hospitals/medical centers just after returning from Saudi Arabia. The patients had fever and/or respiratory symptoms. A summary of the patients’ characteristics is presented in the Table. Of the 7 patients, 4 had cough, 1 had dyspnea, and 4 had fever. Patients 1, 2, and 7 had an acute febrile illness and clinical and/or radiologic evidence of pulmonary parenchymal disease. Patient 1 had sought medical care in Saudi Arabia, and patient 2 had been hospitalized for 10 days in Saudi Arabia.

**Table:** Characteristics of pilgrims who returned from the Hajj with acute respiratory illness and detectable virus in respiratory specimens, Austria, 2014*

Patient no.	Age, y/sex	Other condition(s)	Signs/symptoms	Date of return from Hajj, Oct 2014	Date of sample collection, Oct 2014	Detected respiratory virus
1	50/M	Diabetes	Fever of 38°C, pneumonia, bronchitis	22	23	Influenza B†
2	49/M	Hypothyroidism	Fever of 39°C, pneumonia, bronchitis	21	22	Influenza A(H3N2)‡
3	47/F	None	Fever, cough	19	21	Influenza B†
4	57/M	None	Cough, bronchitis	19	21	Influenza B†
5	66/M	Hypertension, cardiomyopathy	Cough, lung infiltrates	13	20	Rhinovirus
6	54/M	Diabetes, hypertension	Cough, bronchitis	10	13	Rhinovirus
7	52/F	Diabetes, hypertension, bronchial asthma	Fever of 40°C, dyspnea	12	16	Influenza A(H3N2)‡

*All patients were negative for Middle East respiratory syndrome coronavirus. †B/Phuket/3073/2013-like virus, Yamagata lineage. ‡A/Hong Kong/146/2013-like virus.

For the diagnosis of viral infection, a serum sample and a sputum, throat swab, or bronchoalveolar lavage sample were collected and sent to the Department of Virology, Medical University of Vienna, Austria, for analysis. All samples were tested for MERS-CoV by using reverse transcription PCR targeting regions upstream of the envelope gene (*6*). Respiratory and serum samples from all 7 patients were negative for MERS-CoV. The respiratory samples were also tested for influenza A and B viruses and for rhinoviruses, as previously described (*7*–*9*). Of the 7 patients, 3 were positive for influenza B virus, 2 for influenza A(H3N2) virus, and 2 for rhinoviruses (Table). Subsequent phylogenetic analysis showed that the influenza A(H3N2) strains belonged to the A/Hong Kong/146/2013-like viruses and the influenza B strains belonged to the B/Phuket/3073/2013-like viruses of the Yamagata lineage, both of which are subtype H3N2 and B strains included in the 2014–15 seasonal influenza vaccine for the Northern Hemisphere.

Our results showed that MERS-CoV was not detected in any of these patients, and our findings support those from reports investigating illness among 2013 Hajj pilgrims (*2*). Our data indicate that all patients had a respiratory virus infection, and 5 of the 7 patients were infected with influenza virus. Six of the patients had not received seasonal influenza vaccine before traveling; for the seventh patient, influenza vaccine uptake data were not available. In 2014 in Austria, the incidence of influenza-like illnesses was below the epidemic threshold during weeks 40–43: no cases of influenza were reported by the National Influenza Surveillance network, Austria (http://www.influenza.at). Therefore, it is likely that these 7 patients became infected while at the Hajj, as reported for patients during previous Hajj seasons (*10*), or while traveling through international airports.

Our preliminary data indicate that influenza virus infection was the cause of severe respiratory illness in 5 of 7 Austrian pilgrims returning from the 2014 Hajj. Because influenza is a serious and potentially life-threatening illness, seasonal influenza vaccination is recommended for Hajj pilgrims. Accurate and rapid testing for MERS-CoV and other respiratory viruses, including influenza, is essential for identifying persons who may be contagious and for timely and effective antiviral drug treatment and, thus, should be considered for pilgrims with severe respiratory illness.
